# Dexmedetomidine depresses neuronal activity in the subthalamic nucleus during deep brain stimulation electrode implantation surgery

**DOI:** 10.1016/j.bjao.2022.100088

**Published:** 2022-09-09

**Authors:** Corey Amlong, Deborah Rusy, Robert D. Sanders, Wendell Lake, Aeyal Raz

**Affiliations:** 1 Department of Anesthesiology, University of Wisconsin, Madison, WI, USA; 2 University of Sydney, Sydney, Australia; 3 Department of Anaesthetics, Royal Prince Alfred Hospital, Camperdown, NSW, Australia; 4 Department of Neurosurgery, University of Wisconsin, Madison, WI, USA; 5 Department of Anesthesiology, Rambam Health Care Campus, Haifa, Israel; 6 The Ruth and Bruce Rappaport Faculty of Medicine, Technion – Israel Institute of Technology, Haifa, Israel

**Keywords:** deep brain stimulation, dexmedetomidine, microelectrode, Parkinson's disease, subthalamic nucleus

## Abstract

**Background:**

Micro-electrode recordings are often necessary during electrode implantation for deep brain stimulation of the subthalamic nucleus. Dexmedetomidine may be a useful sedative for these procedures, but there is limited information regarding its effect on neural activity in the subthalamic nucleus and on micro-electrode recording quality.

**Methods:**

We recorded neural activity in five patients undergoing deep brain stimulation implantation to the subthalamic nucleus. Activity was recorded after subthalamic nucleus identification while patients received dexmedetomidine sedation (loading – 1 μg kg^−1^ over 10–15 min, maintenance – 0.7 μg kg^−1^ h^−1^). We compared the root-mean square (RMS) and beta band (13–30 Hz) oscillation power of multi-unit activity recorded by microelectrode before, during and after recovery from dexmedetomidine sedation. RMS was normalised to values recorded in the white matter.

**Results:**

Multi-unit activity decreased during sedation in all five patients. Mean normalised RMS decreased from 2.8 (1.5) to 1.6 (1.1) during sedation (43% drop, p = 0.056). Beta band power dropped by 48.4%, but this was not significant (p = 0.15). Normalised RMS values failed to return to baseline levels during the time allocated for the study (30 min).

**Conclusions:**

In this small sample, we demonstrate that dexmedetomidine decreases neuronal firing in the subthalamic nucleus as expressed in the RMS of the multi-unit activity. As multi-unit activity is a factor in determining the subthalamic nucleus borders during micro-electrode recordings, dexmedetomidine should be used with caution for sedation during these procedures.

**Clinical trial number:**

NCT01721460.

Deep brain stimulation of the subthalamic nucleus (STN) is an effective treatment for advanced Parkinson’s disease.^1^, ^2^, ^3^ To achieve optimal clinical results and avoid side effects, when targeting the STN the deep brain stimulation electrode has to be implanted precisely within the dorsolateral STN.^4^^,^^5^ In many centres, electrophysiological mapping of the nucleus is performed using microelectrode recording (MER) to achieve precise localization of the electrode.^6^^,^^7^ During this procedure, microelectrodes are passed through the target nuclei and the neuronal electrical activity is observed and recorded. The surgical team can identify the precise location of the nucleus and its borders according to the typical activity of its neurons.^4^^,^^6^

During the MER mapping procedure, the patient's head is motionless, fixed inside a stereotaxic frame. The procedure may take several hours. Some centres use varying levels of sedation or general anaesthesia to facilitate this surgery.^8^, ^9^, ^10^, ^11^ However, many centres do not routinely use any sedation out of concern that it might change the activity of STN neurons and interfere with the precise MER localization of the electrode.^12^, ^13^, ^14^ It is therefore not unusual for patients to complain of anxiety or discomfort during the operation^8^ when sedatives or anxiolytics are withheld.

Different drugs have been tested for sedation during electrophysiological mapping of the STN, including propofol,^8^^,^^10^^,^^15^ dexmedetomidine,^9^^,^^15^, ^16^, ^17^, ^18^, ^19^, ^20^, ^21^ remifentanil,^8^^,^^22^^,^^23^ midazolam,^10^^,^^22^ ketamine^24^ and volatile anaesthetics.^11^^,^^25^ Reports comparing the clinical outcomes of deep brain stimulation electrode implantation in fully awake patients versus anaesthetised patients have demonstrated conflicting results: some have shown that it is possible to perform the procedure under anaesthesia but with inferior long term outcomes,^26^ while others have not shown a significant difference.^8^^,^^20^ Very few studies report the actual effects of anaesthetic drugs on STN activity and the accuracy of MER guided electrode placement.^11^

Dexmedetomidine is often used in awake neurosurgical procedures.^11^^,^^16^^,^^27^, ^28^, ^29^ Although it takes longer to achieve stable sedation with dexmedetomidine than with propofol or remifentanil, dexmedetomidine produces sedation with minimal respiratory depression and stable haemodynamics (blunted tachycardia and hypertensive responses to stimuli) in neurosurgical patients.^16^^,^^29^, ^30^, ^31^, ^32^

Pilot studies have shown that dexmedetomidine sedation does not impair MER,^27^ while additional research has shown that although high dose dexmedetomidine reduces STN activity, low dose dexmedetomidine does not.^33^ Others conclude that low dose dexmedetomidine (0.1–0.3 μg kg^−1^ h^−1^) does not impair MER, with results based on observations from a neurophysiologist but without a quantitative assessment on MER or report of the clinical outcomes.^9^ Similarly, others conclude that low dose dexmedetomidine (loading dose of 0.7 μg kg^−1^ over 20 min with a subsequent infusion of 0.3–0.5 μg kg^−1^ h^−1^) does not affect MER based on the observation that recognizable STN activity was present during mild sedation, but no comparison to control was made.^27^ Further workers demonstrated that STN identification remained possible, albeit with questionable precision of MER, despite altered firing properties of STN neurons with dexmedetomidine administration.^34^^,^^35^ Thus, the effects of dexmedetomidine on the STN, its potential to impair MER, and any clinical relevance this may have, remain controversial.

To identify whether dexmedetomidine significantly modulates spiking activity in the STN and interferes with accurate MER identification of the optimal target, we compared the activity of STN neurons before, during and after the administration of dexmedetomidine while performing MER for deep brain stimulation placement at a constant electrode location. Unlike prior studies, we used each patient and recording site as their own control, reducing the variability between locations within the STN, hemispheres, individuals, and observers.

## Methods

This prospective cohort study took place at the University of Wisconsin, Madison, WI. The study was approved by the University of Wisconsin IRB (ref. code 2012-0400; National Institutes of Health trial number NCT01721460). All patients scheduled to undergo bilateral STN electrode implantation surgery with MER for the treatment of Parkinson's disease that agreed to participate in the experiment and signed an informed consent were considered candidates for the study unless one of the exclusion criteria was met. Exclusion criteria included hypersensitivity to dexmedetomidine, baseline bradycardia (heart rate below 50 bpm), known or suspected obstructive sleep apnoea, suspected difficult intubation, pregnancy, age under 18 years or over 85 years, and cognitive disability impairing understanding of the experiment or ability to provide informed consent. A data safety monitoring board was appointed at the request of the IRB before starting the study. The board included an anaesthesiologist, a neurosurgeon and an electrophysiologist who were not involved in the study.

### Surgery and microelectrode recording

After signing an informed consent, patients proceeded with a standard deep brain stimulation implantation procedure as practised at University of Wisconsin Hospital as follows: standard ASA monitors as well as a bispectral index (BIS®) monitor were placed upon arrival to the operating suite, followed by placement of a head frame using local anaesthetic and propofol sedation. The patient was then transferred while sedated to receive a computerised tomography (CT) scan. Upon return to the operating suite the patient's propofol sedation was continued through scalp incision and drilling of burr-holes. Propofol sedation was discontinued, and patients were allowed to fully awaken for the MER part of the surgery. In the first patient we used propofol during the switch between hemispheres. For all other patients, we made skin incision and burr-hole for both sides during the initial propofol sedation period. The shortest interval between propofol sedation and study initiation was 41 min and the average was 96.4 (41.8) min.

**Figure 1:**
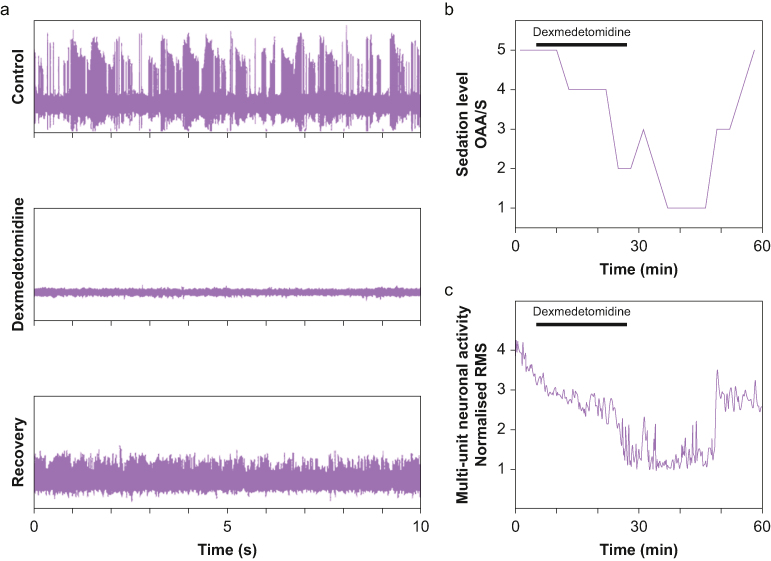
Typical course for a single patient undergoing dexmedetomidine sedation during deep brain stimulation placement. (a) Ten second traces of electrical activity recorded at a single location before dexmedetomidine administration (top), during maximal sedation (middle, OAA/S 1) and during recovery (bottom). (b) Sedation as measured by observer's assessment of alertness/sedation (OAA/S). Dexmedetomidine administration is marked by a grey line at top. (c) Normalised root mean square (RMS) of the multi-unit of activity obtained during dexmedetomidine administration and recovery.

MER was performed using Guideline 4000 system (FHC Inc., Bowdoin, ME) for data acquisition and recording and Medtronic #3389 electrodes. MER started 15–30 mm above the expected STN entry point. During MER the electrodes were advanced in small steps and after each step the neuronal activity was evaluated to allow identification of the STN by its typical neuronal activity (see Fig 1A – top row). A motor examination of the patient was performed periodically by the neurosurgeon. Macrostimulations were used to evaluate the clinical response and possible side effects, followed by permanent stimulating electrode placement and fixation to the skull. Dexmedetomidine administration was performed only on the second of two sides when bilateral deep brain stimulation placement was performed.

### Dexmedetomidine administration

MER was conducted throughout the electrode advancement as part of the procedure. Electrode advancement was stopped following definitive STN identification on the second side. Following three minutes of baseline activity recording at the selected location, dexmedetomidine infusion was started by an anaesthesiologist using a loading dose of 1 μg kg^−1^ over 10–15 min followed by a maintenance infusion of 0.7 μg kg^−1^ h^−1^ until stable sedation was achieved, but no longer than an additional 20 min (to limit the interference with surgery). The dexmedetomidine infusion rate was titrated to achieve the optimal clinical sedation goal. Our goal was to achieve a drowsy but rousable (by voice or a light tap on the shoulder) patient; a score of 3 on the observer's assessment of alertness/sedation (OAA/S) scale.^36^^,^^37^

Following 2–3 min of recordings at the same location under a stable sedation level, we stopped the sedation and allowed the patient to recover. Once the patient was fully awake and cooperative (OAA/S = 4–5) we resumed electrode advancement and MER to identify the ventral border of the STN and completed the electrode implantation as usual.

### Data analysis

We calculated the root mean square (RMS) of the electrical activity as a measure of the multi-unit activity of neurons in the vicinity of the electrode tip. The RMS has been shown to be a useful clinical guide for STN identification and electrode placement.^4^^,^^38^ We normalised the RMS to the baseline value recorded at the first 2–5 min of MER (before entering the STN) to compensate for differences between patients and recording electrodes. To calculate the change in the normalised RMS following sedation we compared the mean RMS obtained during 2 min of stable recording during the baseline (before sedation), during stable sedation and following recovery.

Oscillatory neuronal activity in the beta range (13–30 Hz) is commonly seen in the STN of Parkinsonian patients. The presence of oscillations during electrode placement has been shown to correlate with the success rate of the deep brain stimulation procedure.^5^ We examined the effect of dexmedetomidine sedation on the oscillatory activity in the beta range by comparing the mean beta power in the multi-unit of activity recorded before administrating sedation and during maximal dexmedetomidine sedation.

Descriptive statistics reported in the manuscript are presented as mean and standard deviations (in brackets). As most results did not display normal distribution (as tested by Kolmogorov–Smirnov test), we used the non-parametric Mann–Whitney test for two independent samples to determine the significance of changes. Alpha of 0.05 was considered significant. The time to sedation and recovery was calculated from the clinical data tables recorded during the procedure. Analyses were performed using custom software written in Matlab (MathWorks, Natick, MA).

Based on our previous results using propofol, we calculated that the sample size required to rule out a 15% change in the normalised RMS with alpha of 0.05 and power of 0.8 is 22 patients. We planned an interim analysis of the data following the recruitment of every 5 patients.

## Results

Seven patients were recruited during the years 2013–2015. However, only five underwent sedation: one changed his mind on the day of the procedure, and in another the procedure was aborted before the sedation trial. The protocol was completed in five patients. No adverse events were encountered. Patient characteristics are detailed in Table 1.

**Table 1:** Patient characteristics.

Characteristic	Overall, n = 5
Mean Age, years (range)	60.6 (53–70)
Male/Female, n (%)	2 (40)/3 (60)
Mean Weight, kg (SD)	84 (3.9)
Mean Duration of Symptoms, years (SD)	13.8 (4.7)
Main Indications for deep brain stimulation, n (%)	
Tremor	4 (80)
Rigidity & Postural Instability	1 (20)
Medical Comorbidities, n (%)	
Hypertension	3 (60)
Coronary Artery Disease	1 (20)
Depression	3 (60)
Insomnia	3 (60)
Hypothyroid	1 (20)

Despite the intended study sample size of 22 patients, the study was stopped after the first planned interim analysis following consultation with the data safety monitoring board. The reason for study cessation was the strength of the results – the change in STN multi-unit activity was significant in each patient (even in the patient that achieved only mild sedation). It seemed unnecessary to continue the study as it was clear that even if 17 additional patients were recruited to reach the goal of 22 patients, and in all additional study patients dexmedetomidine failed to cause a significant change (a very unlikely possibility considering the interim results), there would still be a significant enough portion of the population in which MER is affected to not change our final conclusion.

### Sedation

We achieved the sedation goals using our dexmedetomidine regimen within the allocated 20 min in four out of five patients. Patients usually achieved the required sedation level around the time of loading dose completion or shortly thereafter. Figure 1B demonstrates the clinical sedation course of one patient. A single patient was sedated only to OAA/S 4 despite receiving a loading dose over 10 min followed by multiple incremental dose increases. Two others experienced mild over-sedation (OAA/S 1–2) for a few minutes following the loading dose. The average peak BIS value before sedation was 96.0 (2.0) (n = 4, one patient monitor was disconnected before sedation) which dropped to a minimum of 58.6 (19.7) (n = 5) at maximal sedation. Even in this small sample, the fall in BIS was significant (p = 0.029, Mann–Whitney test, n = 4, calculated only for the 4 patients with the complete BIS data).

We experienced no significant bradycardia, hypotension, desaturation, or other clinically significant adverse effects in any of the patients. Recovery from sedation to an OAA/S score of 4–5 ranged from 6 to 32 min, which was expected given the established clinical properties of dexmedetomidine.^39^

### Multi-unit activity

**Figure 2:**
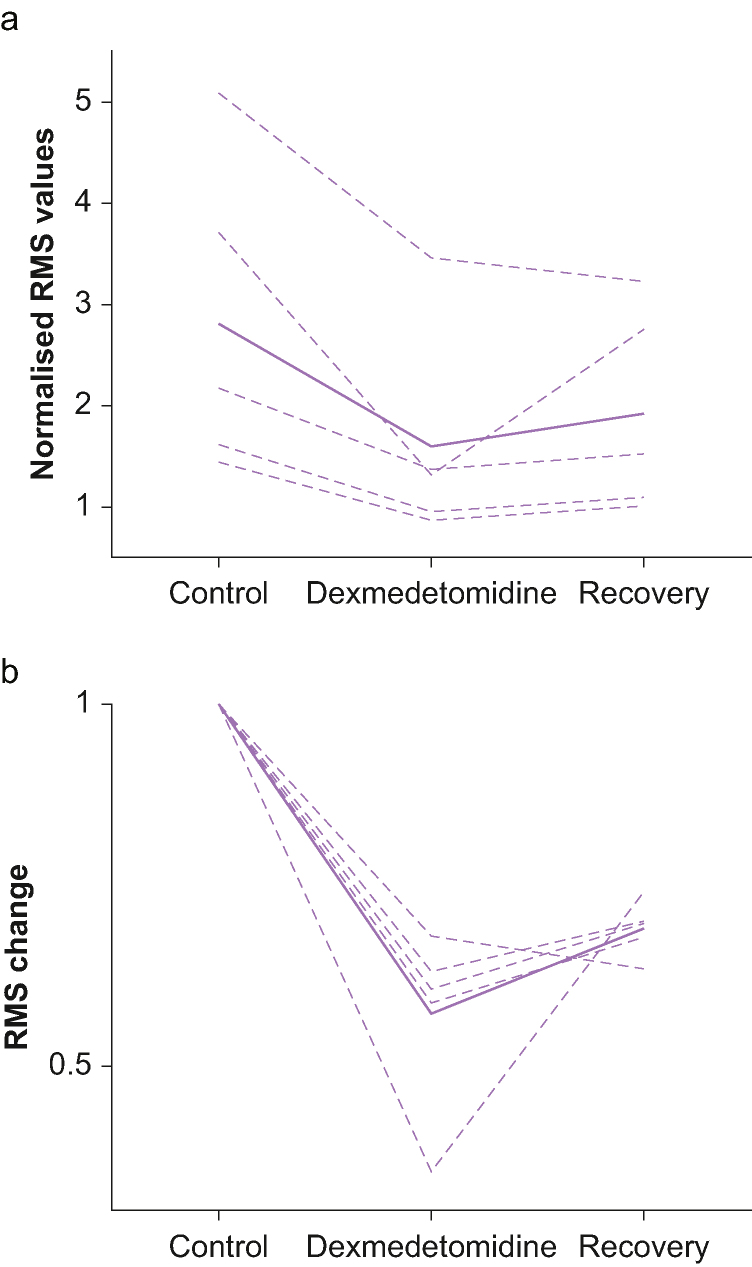
**(a)**: Normalised RMS power of the multi-unit activity before, during and after recovery from dexmedetomidine administration. The dashed lines are single patient results and the solid line is the average. **(b)**: RMS power normalised to the control values to demonstrate the magnitude of the change. It can be seen that there was a substantial drop in RMS power in all patients.

Figure 1A depicts 10 seconds of raw data recorded at the same location before and during dexmedetomidine sedation, as well as after the patient had recovered from sedation. The RMS of the multi-unit of activity was used to evaluate the spiking activity near the tip of the electrode. We normalised this measure to the RMS of the multi-unit of activity in the white matter before entering the STN. To evaluate the effect of dexmedetomidine, we compared the values before starting dexmedetomidine infusion to the values during stable sedation around the time that dexmedetomidine sedation was stopped. In all patients (including the patient in which we achieved only mild sedation) normalised RMS values during sedation were significantly lower than baseline values (Fig 2). It was interesting to find that in two patients the RMS values during sedation were smaller than 1 (that is, RMS values were lower than the values recorded outside of the STN). The mean normalised RMS at baseline was 2.8 (1.5) and decreased to 1.6 (1.1) under dexmedetomidine (43% decrease, p value = 0.056, Mann–Whitney test).

### Oscillatory neural activity

**Figure 3:**
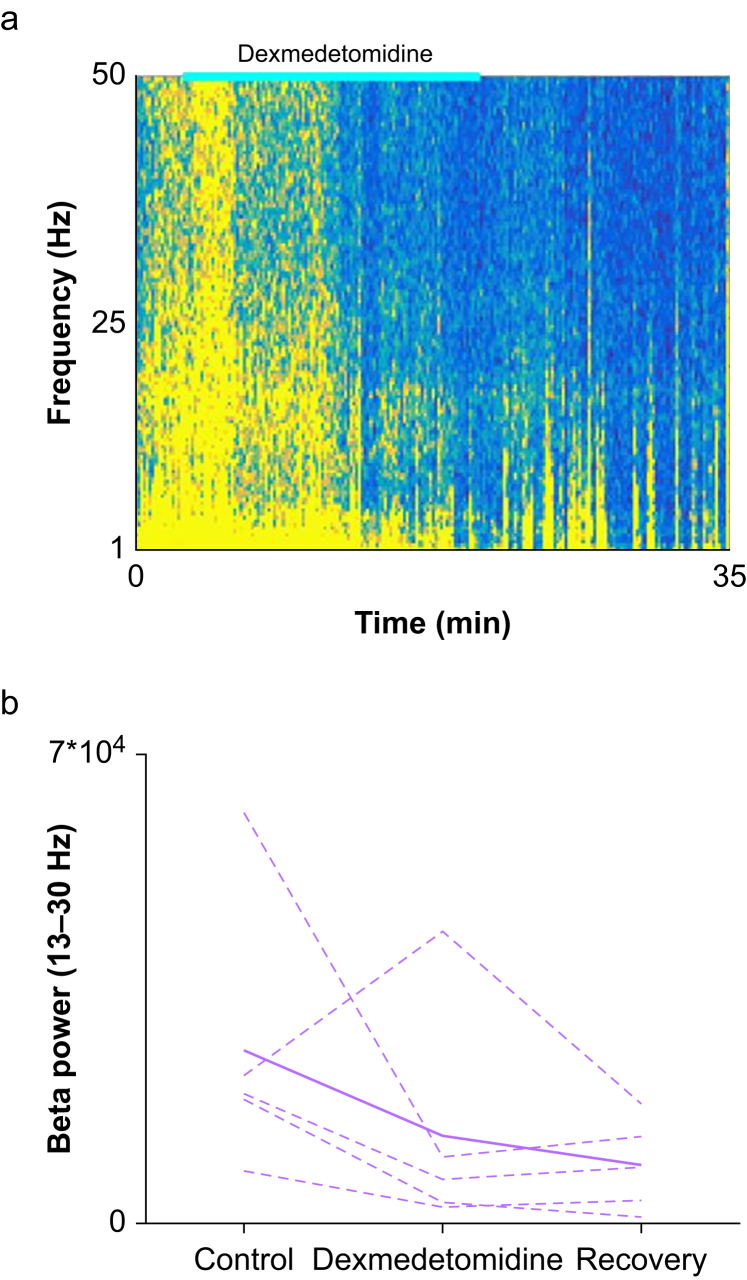
(a): Spectrogram of the neuronal activity before, during and after dexmedetomidine administration. Dexmedetomidine administration is indicated by a bright blue line at the top. (b) Total power in the beta (13–30 Hz) band. The dashed lines are single patient results and the solid line is the average. Interestingly, removing the outlier patient whose beta power increased, brought the decrease to a borderline significance (*P* = 0.057 Mann–Whitney test).

In 4 out of 5 patients we found a decrease in beta power during sedation (Fig 3). Average beta power dropped from 25934 (20348) at baseline to 13386 (17193) during sedation and further to 9007 (6792) after recovery from the dexmedetomidine. However, the decrease was relatively small and in one patient there was an increase. Thus, the overall changes in the mean beta power were not significant (p = 0.15 Mann–Whitney test).

### Recovery of neural activity

Dexmedetomidine was stopped after 16 (3.8) min of administration. To evaluate whether dexmedetomidine would be useful for the initial part of the procedure before initiating MER, we evaluated the recovery of the RMS after sedation was stopped. We calculated the normalised RMS of the multi-unit of activity once the patient had recovered to an OAA/S score of 4–5 before resuming the MER to identify the ventral border of the STN and complete the procedure. The mean normalised RMS during recovery was 1.9 (1.0), a 31% decrease from the baseline value. This was an intermediate value which was not significantly different than either baseline (p value = 0.31, Mann–Whitney test) or sedation values (p value = 0.55, Mann–Whitney test). In no case did the normalised RMS reach the baseline values during the time allocated for the study.

## Discussion

Dexmedetomidine sedation is considered a reliable, effective, and safe option for awake neurosurgical procedures. Under this sedation the patients are comfortable yet alert enough for neurological assessment.^16^^,^^27^, ^28^, ^29^^,^^32^ They maintain intact airway reflexes and spontaneous breathing, while any haemodynamic side-effects of the drug such as lowering of the heart rate and blood pressure are advantageous for these procedures without compromising the patient.^16^^,^^27^, ^28^, ^29^, ^30^ However, dexmedetomidine is known to have slow kinetics. Thus, it takes a long time relative to other sedatives to titrate it to an appropriate depth of sedation, and recovery from this sedation is slow.^30^^,^^32^^,^^39^ Our clinical experience fits well with the known pharmacology of the drug. Two of our patients were initially sedated beyond our goal based on the OAA/S scale, and one patient failed to reach the target sedation level.

Relatively few studies directly examine the effects of dexmedetomidine on the firing rates of STN neurons. Elias and colleagues found that dexmedetomidine decreased STN firing rate only at high doses, and recommended the use of low doses,^33^ whereas Krishna and colleagues reported a slight increase in the firing rate only in the dorsal region of the STN and a significant change in the beta oscillation in the nucleus.^34^ They recommended avoiding this drug mostly because of its effect on oscillations. A more recent study analyzing local field potentials in the STN as measured by in-situ deep brain stimulation electrodes in Parkinson's patients concluded that dexmedetomidine sedation did not alter STN activity at the doses studied (up to 0.6 μg kg^−1^ h^−1^),^21^ however local field potentials are known to be much less sensitive to physiological and pharmacological perturbations than MERs. Another recent retrospective analysis of patients undergoing deep brain stimulation placement under procedural sedation concluded that STN neuronal properties, as measured by MER and multi-unit of activity, were indeed influenced by dexmedetomidine in a dose-dependent manner.^19^

Here we describe a consistent decrease in firing rates during dexmedetomidine sedation. We found that the decreased activity was consistent even when sedation had ceased and the patient was easily rousable, a finding suggested by others.^19^ A notable strength of our study is that we recorded the activity at the same location in the STN before and after administration of dexmedetomidine sedation. Thus, we eliminated the problem of firing rate variability between different patients and locations within the STN and were able to compare sedation and post-sedation recordings to an individual's baseline.

We stopped the study following the first interim analysis after recruiting only five patients because we discovered a substantial effect of dexmedetomidine on the multi-unit of activity in the STN in all subjects. The dramatic decrease in STN activity (in two patients to values lower than the baseline RMS values in white matter) conflicts with previous reports of effective MER during dexmedetomidine administration.^9^^,^^11^^,^^17^^,^^18^^,^^27^ This may be related to the different doses used – we used 1 μg kg^−1^ over 10–15 min followed by a maintenance infusion of 0.7 μg kg^−1^ h^−1^; higher than doses used in other reports, yet a widely used and clinically appropriate dose for procedural sedation. Another possibility is that dexmedetomidine affects not only the activity inside the STN, but also the activity in other areas, thereby altering recordings in the white matter outside of the STN. In such a case, one would still expect to find a change in MER activity when driving the electrode between white matter and the STN without obscuring detection of this border.

Several factors limit the conclusions that can be drawn from this study. Most notably this is a small study, making it difficult to draw conclusions – especially regarding negative results. To ensure that the sedation trial did not affect the clinical results, we recorded only from the second side when patients were already fatigued from a long awake procedure. As our patients were sedated with propofol in the initial stage of the procedure, there is a possibility, albeit unlikely, that residual propofol (long known to effect STN activity^8^) could have altered baseline recordings. Given the substantial delay between propofol cessation and study recording, any such effect would be negligible. Furthermore, the effect of propofol decreases with time. Thus, if there was a measurable propofol effect, we would expect to observe an increase in the RMS during the later stages of the recordings rather than the decrease we observed. It should also be noted that the clinical response to sedation was highly variable among patients receiving the same dose regimen, making comparison difficult using the OAA/S scale. However, in all patients (even those with minimal clinical response) we noticed a significant drop in the RMS. The study design allowed a comparison of neural activity before, during and after sedation at the same location within the STN, effectively making each patient their own control.

In conclusion, our study confirms previous results showing that dexmedetomidine at clinically relevant doses dramatically decreases STN neuronal activity and may interfere with accurate identification of STN borders and precise localization of the stimulating electrode during deep brain stimulation placement. Moreover, it seems that dexmedetomidine has a relatively long-lasting effect on STN firing with a significant delay in return of baseline STN activity after cessation of sedation.

Dexmedetomidine clearly influences MER mapping and STN activity. It is unclear, however, if the derangement of STN activity caused by dexmedetomidine sedation would prevent accurate lead placement and thus negatively affect long-term clinical outcomes. A recent study suggests that this, in fact, is not the case.^20^ Nevertheless, given our findings of significant STN perturbations during dexmedetomidine sedation, it would seem prudent to use this drug with caution during and prior to MER mapping and it is important that the clinicians are aware to the effect it may have on the MER.

## Funding statement

This work was supported by the International Anesthesia Research Society (IMRA to AR) in 2013, the United States—Israel Binational Science Foundation (BSF) Grant 201732 to AR, and the UW Department of Anesthesiology.

## Authors' contributions

Study conception/design: DR, AR.

Data acquisition: all authors.

Data analysis/interpretation: AR, RS.

Drafting of paper: AR, CA.

Critical revision of paper with important intellectual contribution: all authors.

Final approval: all authors.

## Declarations of competing interest

Aeyal Raz reports receiving consultant fees from Medtronic and Neuroindex, and presented a webinar for MSD. All the authors declare that they have no conflicts of interest.
